# Infarct size following complete revascularization in patients presenting with STEMI: a comparison of immediate and staged in-hospital non-infarct related artery PCI subgroups in the CvLPRIT study

**DOI:** 10.1186/s12968-016-0298-2

**Published:** 2016-11-09

**Authors:** Jamal N. Khan, Sheraz A. Nazir, John P. Greenwood, Miles Dalby, Nick Curzen, Simon Hetherington, Damian J. Kelly, Daniel Blackman, Arne Ring, Charles Peebles, Joyce Wong, Thiagarajah Sasikaran, Marcus Flather, Howard Swanton, Anthony H. Gershlick, Gerry P. McCann

**Affiliations:** 1 Department of Cardiovascular Sciences, University of Leicester and the NIHR Leicester Cardiovascular Biomedical Research Unit, University Hospitals of Leicester NHS Trust, Glenfield Hospital, Leicester, UK; 2 Multidisciplinary Cardiovascular Research Centre and The Division of Cardiovascular and Diabetes Research, Leeds Institute of Cardiovascular and Metabolic Medicine, University of Leeds, Leeds, UK; 3 Harefield Hospital, Royal Brompton and Harefield Foundation Trust, NIHR Cardiovascular Biomedical Research Unit, Middlesex, UK; 4 University Hospital Southampton NHS Foundation Trust and University of Southampton, Southampton, UK; 5 Kettering General Hospital, Kettering, NN16 8UZ UK; 6 Royal Derby Hospital, Derby, UK; 7 Leicester Clinical Trials Unit, University of Leicester, UK and Department of Mathematical Statistics and Actuarial Science, University of Leicester, University of the Free State, Bloemfontein, South Africa; 8 Norfolk and Norwich University Hospitals NHS Foundation Trust and Norwich Medical School, University of East Anglia, Norwich, UK; 9 The Heart Hospital, University College London Hospitals, London, UK

**Keywords:** Myocardial infarction, Primary percutaneous coronary intervention, Multivessel disease, Cardiovascular magnetic resonance, Infarct size

## Abstract

**Background:**

The CvLPRIT study showed a trend for improved clinical outcomes in the complete revascularisation (CR) group in those treated with an immediate, as opposed to staged in-hospital approach in patients with multivessel coronary disease undergoing primary percutaneous intervention (PPCI). We aimed to assess infarct size and left ventricular function in patients undergoing immediate compared with staged CR for multivessel disease at PPCI.

**Methods:**

The Cardiovascular Magnetic Resonance (CMR) substudy of CvLPRIT was a multicentre, prospective, randomized, open label, blinded endpoint trial in PPCI patients with multivessel disease. These data refer to a post-hoc analysis in 93 patients randomized to the CR arm (63 immediate, 30 staged) who completed a pre-discharge CMR scan (median 2 and 4 days respectively) after PPCI. The decision to stage non-IRA revascularization was at the discretion of the treating interventional cardiologist.

**Results:**

Patients treated with a staged approach had more visible thrombus (26/30 vs. 31/62, *p* = 0.001), higher SYNTAX score in the IRA (9.5, 8–16 vs. 8.0, 5.5–11, *p* = 0.04) and a greater incidence of no-reflow (23.3 % vs. 1.6 % *p* < 0.001) than those treated with immediate CR. After adjustment for confounders, staged patients had larger infarct size (19.7 % [11.7–37.6] vs. 11.6 % [6.8–18.2] of LV Mass, *p* = 0.012) and lower ejection fraction (42.2 ± 10 % vs. 47.4 ± 9 %, *p* = 0.019) compared with immediate CR.

**Conclusions:**

Of patients randomized to CR in the CMR substudy of CvLPRIT, those in whom the operator chose to stage revascularization had larger infarct size and lower ejection fraction, which persisted after adjusting for important covariates than those who underwent immediate CR. Prospective randomized trials are needed to assess whether immediate CR results in better clinical outcomes than staged CR.

**Trial registration:**

ISRCTN70913605, Registered 24th February 2011.

**Electronic supplementary material:**

The online version of this article (doi:10.1186/s12968-016-0298-2) contains supplementary material, which is available to authorized users.

## Background

The management of multivessel coronary artery disease in patients with ST-segment myocardial infarction at primary percutaneous coronary intervention (PPCI) is controversial (1). Registry data have suggested that a staged complete revascularization (CR) strategy results in better clinical outcomes than immediate CR at the time of PCI. However two recent randomised, controlled trials (2, 3) demonstrated reduced medium-term major adverse cardiovascular event (MACE) rates compared with infarct related artery (IRA)-only revascularization. These findings have resulted in the withdrawal of the American College of Cardiology Choosing Wisely advice of not to undertake CR at the time of PPCI (4). In addition we have shown that CR is not associated with an increase in total infarct size assessed by in-patient cardiovascular magnetic resonance (CMR), despite a small increase in type 4a MI compared to an IRA-only revascularization strategy (5).

There remains however no consensus on whether in-hospital complete revascularisation should be staged (staged CR) or undertaken immediately after PPCI (Immediate CR). In the CvLPRIT study (3), there was a trend for reduced clinical events (death/MI/heart failure) in patients who had immediate (3.1 %) rather than staged (11.9 %) CR.

The aim of this post hoc analysis of the CvLPRIT CMR substudy (5) was to assess infarct size and LV function in patients who underwent immediate compared to staged CR, in order to gain insight into the likely mechanisms to explain the differences in clinical outcomes.

## Methods

### Study design

The study design and main results have been published previously (3, 6). CvLPRIT CMR was a prespecified substudy of a multicenter, prospective, randomized, controlled, open- label, clinical trial with blinded CMR endpoint analysis (PROBE design) conducted in 7 UK centers between May 2011 and May 2014 (5). Inclusion and exclusion criteria were as for the main trial with absolute contraindications to CMR imaging as an additional exclusion.

### Patient recruitment and treatment

**Fig. 1:**
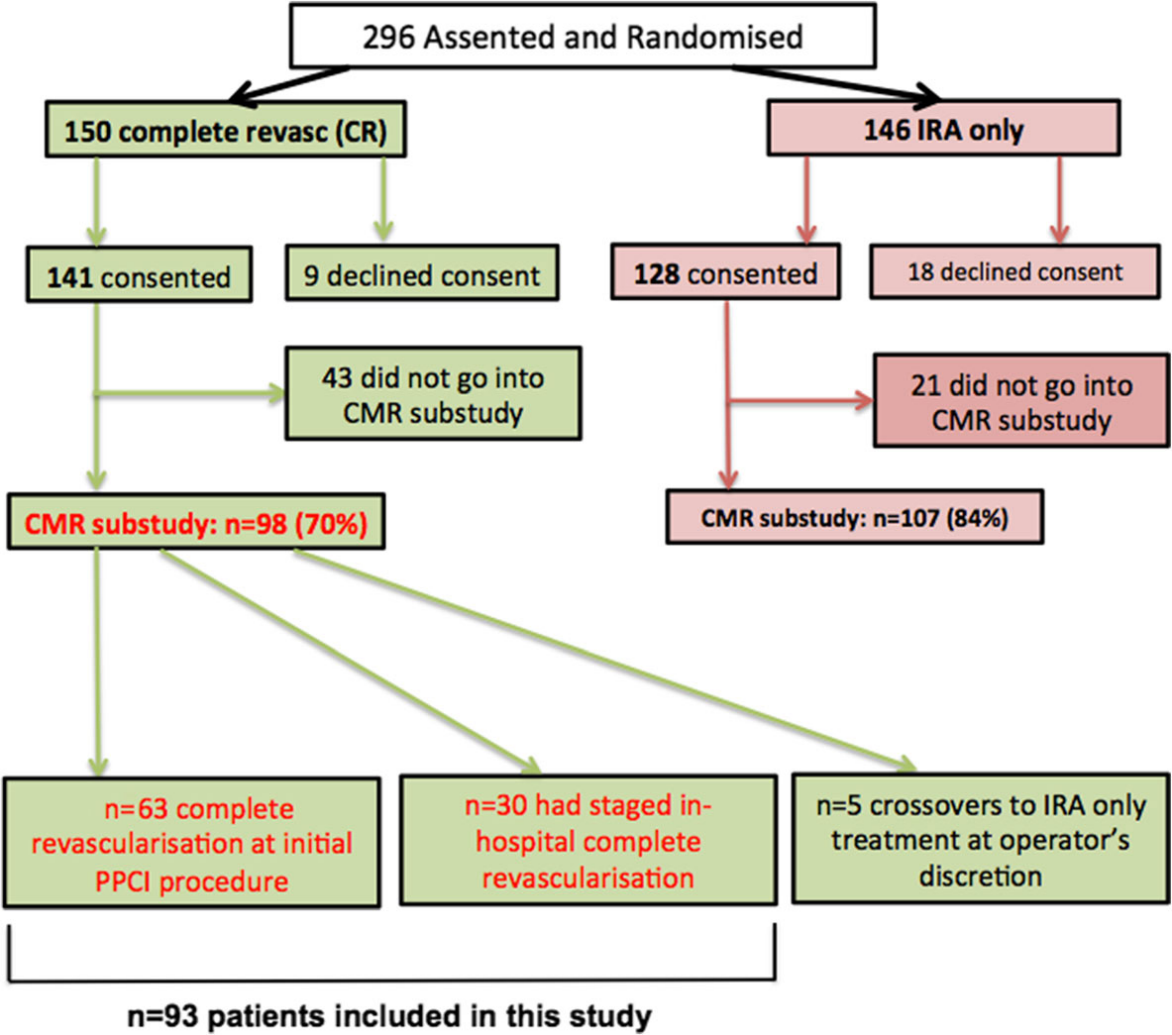
Consort diagram for patient recruitment. CONSORT diagram illustrating recruitment and patient flow. In the topmost green and red boxes are the numbers of patients randomised to each treatment arm (intention to treat) and the number who subsequently received each treatment. CR = complete revascularisation, IRA = infarct related artery; CMR = cardiovascular magnetic resonance

After verbal assent patients were randomized after coronary angiography but before IRA PCI, to IRA-only or in-hospital complete revascularization. If there were no clinical contraindications, immediate CR was recommended but the non-IRA procedure could be staged, at the operator’s discretion, but completed during the index admission. Reasons for staging revascularization were not recorded. Recruitment is shown in Fig. 1. Ninety-eight patients in the substudy were randomised to in-hospital CR, of which 63 were performed immediately and in 30 the procedure was staged. Five patients crossed over into the IRA-only treatment arm.

### Angiographic analysis

Pre and post-PPCI epicardial coronary flow was assessed using Thrombolysis In Myocardial Infarction scoring (7). Collateral flow to the IRA pre-PPCI was graded using the Rentrop system (8). Quantitative Coronary Angiography (QCA) was undertaken using *QAngioXA v1.0* software (Medis, Leiden, Netherlands). Myocardium at risk was angiographically quantified using the Alberta Provincial Project for Outcome Assessment in Coronary Heart Disease (APPROACH score) (9, 10).

### CMR

The CMR methods have been described in detail previously (5). In brief, CMR was performed pre-discharge and after any staged procedure and at 9 months (follow-up CMR).

### Pre-discharge CMR

After localisers and long axis cine images, complete stacks of short axis images covering the entire left ventricle (LV) were acquired with (1) T2w-STIR to determine the area at risk, (2) cine images for LV volumes, mass and ejection fraction and (3) late gadolinium enhanced (LGE) images to determine infarct size and MVO after administration of 0.2 mmol/kg of Magnevist (Bayer, Leverkusen, Germany).

### Follow-up CMR

Follow-up CMR was performed at 9 months (±4 weeks) post-PPCI. The protocol for follow-up CMR was similar to the pre-discharge scan, but with T2w-STIR imaging omitted and assessment of reversible ischemia with first-pass perfusion after pharmacological stress with adenosine included.

### CMR analysis

Analysis was performed as previously described by physicians blinded to all clinical data including treatment allocation at the University of Leicester core lab (5). Briefly, infarct size was quantified on LGE imaging using the Full-Width Half-Maximum technique (11). On the pre-discharge CMR scan, ischaemic area-at-risk (oedema) was assessed using Otsu’s Automated Technique (12) and myocardial salvage index (MSI) was calculated as the percentage of the area at risk that was not infarcted on LGE (5). If infarction was seen in >1 coronary territory in the pre-discharge CMR, this was recorded as being in the IRA territory (associated oedema and/or MVO) or the non-IRA territory with the consensus of three observers (JNK, GPM, JPG). Non-IRA infarcts were additionally classified as likely to be acute or chronic (presence of wall thinning and no oedema/MVO). Infarct size was recorded for both IRA and non-IRA LGE and total infarct size was the sum of all LGE. On the follow-up CMR, perfusion images were visually assessed for defects and reversible ischaemia burden calculated as a percentage expression of the summed difference score (13).

### Clinical outcomes and follow-up

MACE comprised a composite of all-cause mortality, recurrent MI, heart failure and ischemia-driven revascularization. Secondary endpoints included cardiovascular death and individual components of the primary endpoint. Safety endpoints comprised stroke, major bleeding and contrast-induced nephropathy. Data were collected by an independent clinical trials unit (Royal Brompton Hospital, London) and events adjudicated by blinded clinicians.

### Statistical analysis

The primary outcome of the CMR substudy was infarct size (expressed as % of LV mass) on pre-discharge CMR, analysed on a log-transformed scale due to right skew. This was adjusted for known baseline predictors of infarct size (anterior MI, time to revascularization, diabetes, TIMI flow pre-PPCI) and important baseline variables that significantly differed between the two groups (TIMI flow post-PPCI, SYNTAX score, dual antiplatelet therapy choice, glycoprotein inhibitor/bivalirudin use for N-IRA PCI) using generalized mixed models. Normally distributed continuous variables were expressed as mean ± standard deviation and comparison was with student’s t-tests. Non-normally distributed data were expressed as median (25^th^–75^th^ quartiles) and analysed using Mann-Whitney testing. Categorical variables were compared using Chi-squared testing. Clinical outcomes were assessed using time-to-first event survival analysis (log-rank test with right censoring). Kaplan-Meier curves were plotted for the period of randomization to the occurrence of the clinical outcomes and compared using log-rank test, and Cox proportional hazard models were fitted to estimate hazard ratios and 95 % confidence intervals for treatment comparisons.

## Results

### Baseline characteristics

Baseline characteristics and comorbidities were closely matched in the in-hospital staged and Immediate CR subgroups and were similar to those in the overall CvLPRIT study population (Table 1). Four patients in the immediate CR group versus none in the staged group had a history of non-STEMI and previous PCI.

**Table 1:** Baseline characteristics of main CvLPRIT trial and immediate versus staged in-hospital complete revascularisation CMR substudy participants

Variable	CvLPRIT cohort (*n* = 296)	Immediate CR (*n* = 63)	Staged CR (*n* = 30)	*p*
Age (y)	64.9 ± 11.6	63.0 ± 11.6	65.0 ± 10.3	0.42
Male sex (%)	240/296 (81.1)	55 (87.3)	28 (93.3)	0.38
BMI (kg/m^2^)	27.3 (24.4–30.2)	27.7 ± 4.5	27.6 ± 4.1	0.95
Heart rate (beats per minute)	74.4 ± 17.6	71.9 ± 16.4	73.5 ± 18.0	0.68
Systolic BP (mmHg)	137.6 ± 27.1	132.6 ± 26.8	140.0 ± 27.7	0.23
Anterior infarct (%)	106 (35.6)	21 (33.3)	11 (36.7)	0.75
eGFR (ml/min/1.73)	95.74 ± 34.7	96.1 ± 30.2	101.5 ± 41.0	0.49
Hypertension (%)	105/287 (36.6)	24 (38.1)	10 (33.3)	0.66
Hypercholesterolemia (%)	75/287 (26.1)	16 (25.4)	12 (40.0)	0.15
Diabetes Mellitus (%)	39/287 (13.6)	11 (17.5)	4 (13.3)	0.61
Current smoker (%)	87/285 (30.5)	23 (36.5)	10 (33.3)	0.77
Previous MI (%)	12/287 (4.2)	4 (6.3)	0 (0.0)	0.16
Previous PCI (%)	9/287 (3.1)	4 (6.3)	0 (0.0)	0.16
Anti-anginal medication (B/N)	54/287 (18.8)	8/63 (12.7)	5/29 (17.2)	0.56
Killip Class II-III (%)	24/286 (8.4)	4 (6.3)	2 (6.7)	0.95

*Abbreviations*: *CR* complete revascularization, *BME* black or minority ethnicity, *BMI* body mass index, *eGFR* estimated glomerular filtration rate, *CK* creatine kinase, *MI* myocardial infarction, *PCI* percutaneous coronary intervention

Anti-anginal medication (B/N) = beta-blocker or nitrate at admission

### Angiographic and PCI details

The median time to staged non-IRA PCI was 34.2 h post-PPCI (IQR 24.8–48.9). There was increased visible thrombus, subsequent thrombectomy catheter use, a higher incidence of IRA no-reflow and reduced TIMI grade post-PPCI in staged CR patients (Table 2). There was a small but significant increase in CAD complexity in the staged group (SYNTAX score 18.3 vs. 16, *p* = 0.021) involving the IRA (*p* = 0.043). The prevalence of well collateralised IRA territory and LAD IRA were similar in both groups. The angiographically derived AAR on APPROACH score was similar in the groups. Patients with right coronary artery IRA were more likely, and those with circumflex IRA less likely, to have a staged procedure. There was less glycoprotein IIb/IIIa inhibitor and bivalirudin use during the non-IRA PCI in the staged compared to the immediate CR group. When the staged and PPCI procedures were added, there was significantly increased cumulative screening time, contrast dose, number of stents (non-IRA PCI and total number of stents) and total procedure lengths in staged versus immediate CR (Table 2).

**Table 2:** Periprocedural details in the immediate and staged in-hospital complete revascularisation groups

Variable	Immediate CR (*n* = 63)	Staged CR (*n* = 30)	*p*
Symptom to PCI time (min)	180 (128–307)	203 (152–296)	0.95
Radial access (%)	50 (80.6)	27 (90.0)	0.26
Aspirin	62 (98.4)	30 (100)	0.49
Second antiplatelet agent (*n*, %)	63 (100)	30 (100)	1.00
GPI during PPCI (*n*, %)	20 (31.7)	11/29 (37.9)	0.56
Bivalirudin during PPCI (*n*, %)	32 (53.3)	17/27 (63.0)	0.40
*Infarct related artery*:			
Left Anterior Descending (*n*, %)	20 (31.7)	11 (36.7)	0.64
Right Coronary (*n*, %)	24 (38.1)	19 (63.3)	0.022
Circumflex (*n*, %)	19 (30.2)	0 (0)	0.001
Visible thrombus (*n*, %)	31/62 (50.0)	26/30 (86.7)	0.001
Thrombectomy catheter (%)	39/63 (61.9)	26/30 (86.7)	0.015
Vessels with ≥75 % stenosis (*n*)	1.5 ± 0.6	1.6 ± 0.6	0.82
Stenosis in non-IRA lesions (%)	73.4	72.9	0.85
SYNTAX score (total)	16 (12–21.5)	18.3 (15–26)	0.021
SYNTAX score (IRA)	8 (5.5–11)	9.5 (8–16)	0.043
SYNTAX score (NIRAs)	6 (4–9)	7 (4.8–12)	0.24
Rentrop grade	0 (0–1)	1 (0–1)	0.35
Rentrop grade 2–3 pre PCI (*n*, %)	7/63 (11.1)	3/30 (10.0)	0.87
APPROACH area at risk (%)	26.0 ± 11.7	29.2 ± 10.8	0.21
TIMI grade pre PCI	0 (0–1)	0 (0–0)	0.47
TIMI grade post PCI	3 (3–3), 2.92 ± 0.4	3 (3–3), 2.77 ± 0.5	0.023
IRA no-reflow (*n*, %)	1 (1.6)	7 (23.3)	<0.001
GPI at NIRA PCI (*n*, %)	20 (31.7)	4 (7.7)	0.06
Bivalirudin during NIRA PCI (*n*, %)	32/60 (53.3)	3/28 (10.7)	<0.001
GPI or Bivalirudin at NIRA PCI (*n*, %)	50/60 (87.7)	7/28 (25.0)	<0.001
Total Contrast dose (ml)	295 (213–350)	390 (266–555)	0.002
Total Screening time (min)	15.5 (12–21)	21 (17–43.3)	0.001
Total Procedure length (IRA + NIRA, min)	58 (38.5–72.8)	91 (67–154.3)	<0.001
IRA PCI procedure length (min)	53 (35–70.5)	55 (37.5–81.3)	0.08
Total number of stents (*n*)	2.8 ± 1.1	3.4 ± 1.4	0.034
Number of stents in IRA (*n*)	1.3 ± 0.6	1.6 ± 0.8	0.09
Number of stents in NIRAs (*n*)	1.5 ± 0.8	1.8 ± 1.0	0.026

Data presented as *n*/*N* (%), mean ± SD or median (IQR)

*Abbreviations*: *CR* complete revascularization, *IRA* infarct related artery, *PCI* percutaneous coronary intervention, *GPI* glycoprotein IIa/IIIb inhibitor, *QCA* quantitative coronary angiography, *TIMI* thrombolysis in myocardial infarction

### CMR data

#### Pre-discharge CMR

Results are displayed in Table 3. Pre-discharge CMR was undertaken later in staged CR patients than in those undergoing immediate CR (4.1 [2.7–5.4] days post PPCI vs. 2.3 days [1.7–3.2], *p* < 0.001). LV ejection fraction was significantly lower in staged patients. Median total infarct size was significantly greater in staged patients (19.7 % (11.7–37.6) vs. 11.6 % (6.8–18.2) LVM, *p* = 0.016) and this was associated with an increase in peak creatine kinase of borderline statistical significance. When corrected for important covariates, infarct size remained greater (*p* = 0.012). In 22 patients (24 %), area at risk could not be quantified. MSI was lower in staged CR patients and there was a greater extent of MVO (*p* = 0.032).

**Table 3:** Peak creatine kinase and pre-discharge and follow-up CMR data

Variable	Immediate CR (*n* = 63)	Staged CR (*n* = 30)	*p*
Peak CK (IU/L)	939 (627–1567)	1508 (938–2280)	0.05
Pre-discharge CMR			
Total Infarct Size (% LVM)	11.6 (6.8–18.2) 13.5 ± 11.4	19.7 (11.7–37.6) 22.6 ± 14.5	0.016 (0.012)*
Time from PPCI (days)	2.3 (1.7–3.2)	4.1 (2.7–5.4)	<0.001
Infarct on LGE (%)	60 (95.2)	30 (100)	0.22
Patients with >1 acute infarct	7 (11.1)	9 (30.0)	0.024
IRA Infarct size (% LVM)	11.1 (5.4–17.4) 12.5 ± 10.0	19.1 (8.8–35.2) 20.9 ± 14.6	0.039 (0.05)*
Non-IRA Infarct size (% LVM)	0.9 ± 3.2	1.7 ± 3.6	0.11 (0.65)*
Total acute infarcts (% LVM)	11.6 (6.8–17.6) 13.0 ± 10.3	19.1 (10.2–37.1) 21.7 ± 14.8	0.006 (0.025)*
Area at risk (% LVM)	31.4 ± 12.5	33.1 ± 10.8	0.57
MSI^§^ (%)	61.7 (37.4–75.5)	35.1 (5.9–66.4)	0.008 (0.034)*
MVO present (*n* %)	34/63 (54.0)	21/30 (70.0)	0.14
MVO (% LVM)	0.07 (0.00–0.93)	0.44 (0.00–6.1)	0.032 (0.024)*
LVMI (g/m2)	52.5 (47.7–61.0)	51.5 (45.6–63.0)	0.55
LVEDVI (ml/m2)	89.9 (78.4–110.0)	89.7 (82.8–102.9)	0.43
LVEF (%)	47.4 ± 9.4	42.2 ± 10.2	0.019
Follow-up CMR	*n* = 53	*n* = 26	
LVMI (g/m2)	45.2 (38.8–52.3)	47.4 (40.9–51.6)	0.71
LVEDVI (ml/m2)	92.5 (80.5–105.5)	93.9 (83.3–113.6)	0.28
LVEF (%)	50.9 ± 9.4	46.7 ± 8.9	0.06
Infarct on LGE (*n*,%)	51 (96.2)	26 (100)	0.32
Patients with >1 infarct (%)	9 (17.0)	9 (34.6)	0.08
IS (% LVM)	5.7 (2.4–10.4)	13.5 (4.6–23.3)	0.004 (0.044)*

Data presented as *n*/*N* (%), mean ± SD or median (IQR)

*Abbreviations*: *CR* complete revascularization, *IRA* infarct related artery, *LVMI* left ventricular mass index, *LVEDVI* left ventricular end-diastolic volume index, *LVEF* left ventricular ejection fraction, *LGE* late gadolinium enhancement, *IS* infarct size, *MVO* microvascular obstruction, *MSI* myocardial salvage index

^§^Analyzable oedema imaging available in 76 % of patients in both groups

*Adjusted for known predictors of IS (anterior MI, time to revascularization, diabetes, TIMI flow pre-PPCI) and important baseline variables significantly varying between the two groups (TIMI flow post-PPCI, SYNTAX score, dual antiplatelet therapy choice, glycoprotein inhibitor/bivalirudin use for N-IRA PCI)

**Fig. 2:**
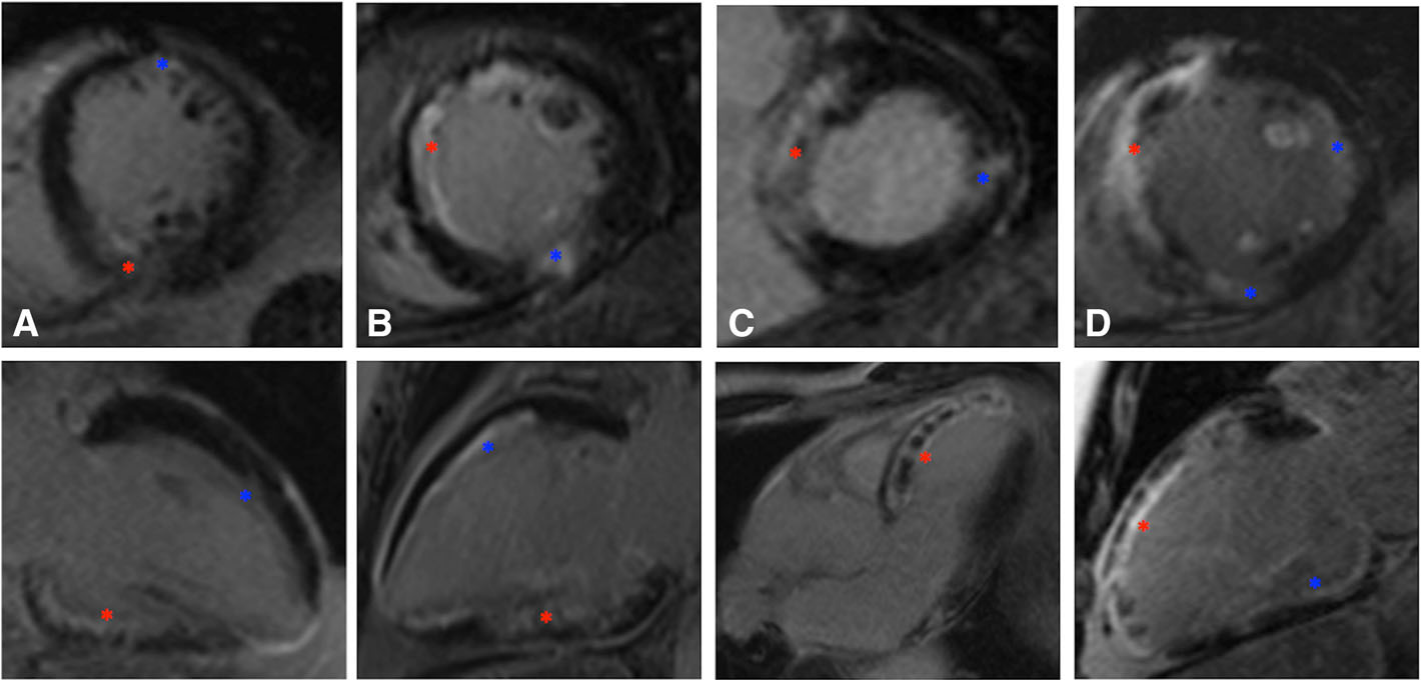
Examples of patients with >1 ‘acute’ MI on CMR. Late gadolinium enhanced short axis (*top row*) and long axis (*bottom row*).  IRA-related infarct;  NIRA-related infarct(s). **a** (X511 Immediate CR): IRA (RCA) inferior infarct 19.1 % LVM, NIRA (LAD) anterior infarct 3.8 % LVM, total IS 22.9 % LVM. **b** (X695 Immediate CR): IRA (RCA) inferior infarct 7.8 % LVM, NIRA (LAD) anteroseptal infarct 5.0 % LVM, total IS 12.8 % LVM. **c** (X757 Staged CR): IRA (LAD) anteroseptal infarct 20.8 % LVM, NIRA (LCX) lateral infarct 0.6 % LVM, total IS 21.4 % LVM. **d** (X798 Staged CR): 3 acute infarcts, IRA (LAD) anteroseptal infarct 35 % LVM, NIRA-1 (RCA) inferior infarct 0.7 % LVM, NIRA-2 (LCX) lateral infarct 2.0 % LVM, total IS 37.6 % LVM. IRA infarct size and non-IRA PCI in Additional file 1: Table S1

The prevalence of non-IRA territory infarcts in staged patients was almost three-fold that of the Immediate CR group (40 % vs. 14 %, *p* = 0.006), including when only acute non-IRA infarcts were included (30 % vs. 11 %, *p* = 0.024). Examples are shown in Fig. 2 and the location, size of infarct, expected coronary artery territory and additional non-IRA PCI are shown in Additional file 1: Table S1. Non-IRA territory infarcts varied considerably in size from 0.1 to 11.9 % of LV mass and averaged 3.7 % (immediate) and 2.9 % (staged) of LV mass. Two patients (3 %) in the immediate and three (10 %) in the staged CR group had chronic non-IRA infarcts (evidenced by wall thinning). Excluding these patients from the analysis did not significantly alter the results (Additional file 1: Table S2).

#### Follow-up CMR

Results are shown in Table 3. Fifty-three patients in the immediate group and 26 in the staged group underwent follow-up CMR. There were no differences in baseline characteristics or pre-discharge CMR findings between those who did and did not attend the follow-up CMR (data not shown). Total infarct size remained greater in staged CR patients (13.5 % vs. 5.7 %, *p* = 0.004, corrected *p* = 0.044). Reversible perfusion defects were seen in 20 % of the immediate and 27 % of the staged patients but the overall ischemic burden was small (2.6 ± 6.9 and 5.2 ± 12.1 % respectively) and not significantly different between groups.

#### Clinical outcomes

Discharge medication was similar between groups (Additional file 1: Table S3). Median follow-up was 365 days (immediate CR 365 days, staged CR 361 days, *p* = 0.75). Length of inpatient stay was longer in staged CR (4.2 ± 3.2 vs. 3.1 ± 1.9, *p* = 0.002) compared to immediate CR. The overall MACE rate was low (6.5 %) at 1 year. The incidence of in-hospital clinical events, overall MACE and individual components were similar in the treatment arms (Additional file 1: Table S4), apart from a higher frequency of major bleeds in staged CR (10.0 % vs. 0.0 %, *p* = 0.011).

## Discussion

This post hoc analysis of patients in the CvLPRIT CMR substudy is the first report of infarct size following immediate and staged CR for multivessel disease at PPCI. We have shown that patients in the CvLPRIT study who were randomized to CR, and in whom experienced interventional cardiologists chose to stage non-IRA PCI, had more visible IRA thrombus, slightly but significantly higher SYNTAX score, lower TIMI scores and more no-flow after PPCI. These differences in baseline angiographic and PPCI results were associated with larger infarcts, less myocardial salvage and reduced ejection fraction compared to patients who had immediate CR. It is important to highlight that patients in this analysis were not randomized to immediate or staged CR and there were many differences in baseline characteristics between the groups. Therefore, despite adjusting for known baseline predictors of infarct size and other variables that significantly differed between the two groups, the results are still likely to suffer from unknown biases and we cannot conclude that staging results in larger infarcts than immediate CR. These data can therefore be considered hypothesis-generating only, but warrant further investigation in larger studies.

### Infarct size, MVO and myocardial salvage

The lower total infarct size and MVO extent, higher MSI and LV ejection fraction observed with immediate CR may be due to a number of possible factors. There could be real differences arising from treatment strategies; the staged group may have been having larger infarcts and thirdly the decision to stage the procedure, at least in some cases, may have been as a direct result of poor technical success e.g. no-reflow of the IRA. We think it is unlikely that staged patients were having larger infarcts at baseline as the time to presentation, proportion having anterior MI, degree of collateralization of the IRA and Killip Class were not significantly different from the immediate CR group and adjusting for these variables did not significantly alter the results. In addition, the ischaemic area at risk was not significantly different in the two groups. This was the case both when quantified on CMR and on the angiographically derived APPROACH score, which would negate any effect of differing CMR timing. A significant effect of ischemic preconditioning is also unlikely given the low prevalence of anti-anginal medication use in both groups (14).

Immediate CR to non-IRA’s could theoretically reduce infarct size by increasing collateral flow or by improved blood flow to the watershed region of the infarct (15). The severity of the non-IRA lesions (average stenosis diameter 73 % in both groups) also indicates that these were likely to have been flow-limiting stenoses. In support of a real effect of immediate CR is the increase in MSI compared to staged patients. However, and most importantly, differences in angiographic and PPCI results most likely explain the reductions in MSI and increased infarct size in the staged v immediate CR groups. The staged group had significantly more visible thrombus in the IRA (87 % v 50 %), subsequent thrombectomy catheter use and significantly more no-reflow (23 % v 2 %) than the immediate CR group. These factors are likely to be the main reason for the increase in infarct size, reduced salvage and decreased ejection fraction. We did not prospectively record the operators’ reasons for staging the non-IRA procedures in staged patients but we think it is likely that a suboptimal result from the PPCI and the presence of inferior rather than lateral MI influenced the decision to stage the non-IRA PCI.

### Non-IRA MI

A surprising finding in this study was that the frequency of non-IRA MI detected by CMR was considerably higher in the staged versus immediate CR groups. PCI related MI (type 4a) are well recognized, (16, 17) although of uncertain clinical significance. In elective PCI patients up to 29 % (18) will have significant increases in troponin and a similar proportion of patients undergoing complex PCI will have evidence of type 4a MI on CMR, even when pre-treated with clopidogrel for >24 h and a glycoprotein IIb/IIIa inhibitor periprocedurally (18). Excluding those patients with evidence of chronic infarction, acute non-IRA MI was seen in 30 % of the staged and only 11 % of the immediate CR groups. Although these type 4a MI were relatively infrequent and small (3.7 and 2.8 % of LV mass for immediate and staged patients respectively) there was considerable variation in size. Revascularization related injury accounting for 4 % of LV mass has been associated with a three-fold increase in MACE (19). Larger randomized studies are required to confirm whether staging CR results in more frequent non-IRA MI and poorer outcomes than immediate CR.

The explanation for the increase in type 4a MI seen with staged CR is likely to be related to greater number of stents implanted in the non-IRA of the staged patients and possibly the different use of adjunctive medication at the time of the non-IRA PCI. Glycoprotein IIb/IIIa inhibitor (8 %) and bivalirudin (11 %) use was low in the staged procedures compared to the immediate CR group (32 and 53 % respectively), which probably reflects clinicians concerns about bleeding with a second in-patient procedure requiring additional vascular access.

### Clinical outcomes

The clinical event rate in both groups was similar (immediate 6.3 % and staged 6.7 %) and lower than seen in the main trial for those randomized to CR (10 %). The lack of other significant differences between the two groups in this post-hoc analysis with small numbers mean no conclusions can be drawn. Immediate CR was associated with a shorter inpatient stay of one night compared with staged CR. This finding and the reduction in lab time with second procedures may suggest that an immediate CR is likely to be more cost effective than a staged strategy (20). However these findings could simply be related to the fact the staged patients had larger MI and although cost-effectiveness will be assessed in the entire CVLPRIT population, any differences between staged and immediate CR would have to be confirmed in randomized trials comparing these strategies. The increased frequency of major bleeds with staged CR is likely secondary to the need for two separate procedures and hence two arterial punctures. However, due to the small numbers, this should be confirmed in a larger study.

This is a post-hoc analysis and patients were not randomised to immediate or staged CR. We did not systematically record the reasons for staging the procedure or use of adjunctive medication, which is a significant limitation. The marked differences in angiographic appearances at baseline, and success following PPCI, are likely to contribute to the observed differences in infarct size between the immediate and staged CR groups. However statistical significance persisted after correction for important baseline covariates. Due to the small numbers of patients in this analysis, propensity matching was not possible. The study was not powered for clinical outcomes. Inevitably, patients who died early or who were very ill following PPCI could not participate in the CMR study which likely explains why the clinical event rates are lower than in the main study. The pre-discharge CMR was undertaken later in staged patients (day 4), which is likely to have resulted in a decrease in infarct size and MVO extent compared with scanning at day 2 (21). Hence, the observed differences in CMR outcomes in immediate and staged CR may have been even greater if both groups were scanned at the same timepoint. However, it was important that the CMR was performed after the staged non-IRA procedures to ensure that we captured associated type 4a MI in our results. Finally, as it was not routinely captured, we could not confirm whether the higher incidence of no-reflow in the staged patients was reflected in less ST-segment elevation resolution post PPCI.

## Conclusions

Patients with staged CR in the CvLPRIT CMR substudy had more visible thrombus in the IRA, higher SYNTAX score, more stents inserted, higher incidence of no-flow and subsequently larger infarct size and reduced ejection fraction, that persisted after correction for important confounders, than patients treated with immediate CR. Prospective randomized trials are needed to assess whether immediate CR results in better clinical outcomes than staged CR.

## Additional file

Additional file 1: Supplemental data. **Table S1**. Patients with 2 or more ‘acute’ MI. **Table S2**. CMR data excluding patients with chronic infarcts on the pre-discharge scan. **Table S3.** Discharge medication. **Table S4.** Clinical outcomes. (DOCX 80 kb)

## Abbreviations

AAR: Area-at-risk
CMR: Cardiovascular magnetic resonance
CR: Complete revascularisation
CvLPRIT: Complete versus Lesion-only Primary PCI Trial
IRA: Infarct related artery
LGE: Late gadolinium enhanced
LV: Left ventricular
MSI: Myocardial salvage index
PPCI: Primary percutaneous coronary intervention
STEMI: ST-Segment elevation myocardial infarction
TIMI: Thrombolysis in myocardial infarction
