# Supplementary material for: Infarct size following complete revascularization in patients presenting with STEMI: a comparison of immediate and staged in-hospital non-infarct related artery PCI subgroups in the CvLPRIT study
Source: J Cardiovasc Magn Reson. 2016 Nov 9;18:85. doi: 10.1186/s12968-016-0298-2 (PMC5109831; doi:10.1186/s12968-016-0298-2)
Supplement: Additional file 1: — Supplemental data. Table S1. Patients with 2 or more ‘acute’ MI. Table S2. CMR data excluding patients with chronic infarcts on the pre-discharge scan. Table S3. Discharge medication. Table S4. Clinical outcomes. (DOCX 80 kb) [file 12968_2016_298_MOESM1_ESM.docx]

## Supplemental data

## Supplemental Table 1: Patients with 2 or more ‘acute’ MI

| **ID** | **No. of MI** | **IRA** | **MI area** | **IRA IS % LV** | **MI 2**  **Area** | **IS 2 % LV** | **IS 3 Area** | **IS 3 %LV** | **CMR**  **N-IRA** | **Actual N-IRA PCI** |
| --- | --- | --- | --- | --- | --- | --- | --- | --- | --- | --- |
| ***Immediate CR*** | | | | | | | | | | |
| X511 | 2 | RCA | I | 19.09 | Apical | 3.82 | -- | -- | LAD | LAD |
| X594 | 3 | RCA | I | 4.27 | AL | 0.94 | A | 0.10 | LCX +LAD | LCX +LAD |
| X599 | 3 | LCX | L | 9.82 | I | 8.93 | AS | 0.98 | RCA + LAD | RCA + LAD |
| X612 | 2 | LAD | A | 42.20 | I | 4.37 | -- | -- | RCA | RCA |
| X665 | 2 | LCX | L | 24.13 | I | 4.35 | -- | -- | RCA ^¶^ | LAD |
| X695 | 2 | RCA | I | 7.75 | AS | 5.01 | -- | -- | LAD | LAD |
| X747 | 2 | LAD | AS | 22.12 | I | 0.37 | -- | -- | RCA | RCA |
| X788 | 2 | LAD | AS | 4.73 | IL | 2.08 | -- | -- | LCX | LCX |
| Mean |  |  |  |  |  | 3.73 |  | 0.54 |  |  |
| ***Staged CR*** | | | | | | | | | | |
| X517 | 2 | LAD | AS | 13.70 | AL | 0.93 | -- | -- | Diag | Diag |
| X530 | 2 | RCA | I | 25.94 | AS | 0.06 | -- | -- | LAD | LAD |
| X540 | 2 | RCA | I | 9.34 | L | 2.82 | -- | -- | LCX | LAD **^§^** |
| X545 | 2 | LAD | AS | 1.56 | IL | 5.19 | -- | -- | LCX | LCX |
| X757 | 2 | LAD | AS | 20.83 | L | 0.60 | -- | -- | LCX | LCX |
| X785 | 2 | RCA | IL | 37.45 | AS | 0.15 | -- | -- | LAD | LAD |
| X791 | 2 | LAD | AS | 7.06* | IL | 11.86 | -- | -- | LCX | LCX |
| X798 | 3 | LAD | AS | 34.87 | I | 0.66 | L | 2.03 | RCA +LCX | RCA +LCX |
| X808 | 2 | LAD | AS | 49.30 | IL | 2.85 | -- | -- | LCX | LCX |
| Mean |  |  |  |  |  | 2.79 |  |  |  |  |

Abbreviations: AL=Anterolateral; AS Anteroseptal; I=inferior; IL= Inferolateral; Diag= Diagonal vessel; Timing of N-IRA PCI: IMMEDIATE= One-time (performed at same sitting as PPCI), Staged: deferred at operator’s discretion to delayed inpatient N-IRA PCI.

*AS MI associated with MVO and therefore classed as the IRA. **^§^** LAD PCI crossed Diagonal; ^¶^ Co-dominant system. CMR Non-IRA probably related to IRA PCI although inferior MI appeared separate from Inferolateral MI with extensive MVO: see Figure 2.

## Supplemental Table 2: CMR data excluding patients with chronic infarcts on the pre-discharge scan

| **Variable** | **One-time CR** | **Staged CR** | **p** |
| --- | --- | --- | --- |
| **Acute CMR** | **n=61** | **n=27** |  |
| Total IS (% LVM)  Median (IQ range)  Mean±SD | 11.6 (6.1-16.1)  [12.4±9.9] | 19.1 (12.2-19.1)  [22.7±14.9] | **0.012**  ***(0.021)**** |
| Time from PPCI (days) | 2.7 (1.8-3.4) | 4.1 (2.7-5.2) | **0.016** |
| Infarct on LGE (%) | 58/61 (95.1) | 27/27 (100) | 0.24 |
| Patients with >1 infarct (%) | 7/61 (11.5) | 9/27 (33.3) | **0.014** |
| IRA Infarct size (% LV Mass)  Median (IQ range)  Mean±SD | 10.5 (4.9-14.9)  11.9±9.5 | 19.1 (9.3-36.2)  21.7±14.8 | **0.006**  ***(0.050)**** |
| NIRA-related IS (% LV Mass) in those with > 1 infarct  Median (IQ range)  Mean±SD | 4.4 (2.1-5.0)  4.4±2.8 | 3.0 (0.4-4.0)  2.7±1.9 | 0.44  ***(0.52)**** |
| Acute MSI (%) | 62.7 (39.4-76.2) | 35.2 (4.6-67.6) | 0.007  ***(0.023)**** |
| Final MSI (%) | 87.0 (70.0-91.1) | 64.3 (46.5-83) | 0.005  ***(0.06)**** |
| **Follow-up CMR** | **n=51** | **n=23** |  |
| Time to CMR (months) | 9.3 (9.0-9.9) | 9.4 (9.1-10.5) | 0.65 |
| Infarct on LGE (n,%) | 49/51 (96.1) | 23/23 (100) | 0.34 |
| Patients with >1 infarct (%) | 7/51 (13.7) | 6/23 (26.1) | 0.20 |
| Total IS (% LVM) | 4.9 (2.3-9.7)  7.2±8.0 | 13.5 (5.8-24.3)  16.6±13.9 | **<0.001**  ***(<0.040)**** |
| NIRA IS (total, % LVM) | 0.00 (0.0-0.0)  1.5±4.4 | 0.00 (0.00-1.5)  1.5±3.6 | 0.98  ***(0.65)**** |
| **Perfusion** | **n=49** | **n=23** |  |
| Ischaemic burden (%)* | 2.6±7.0 | 3.7±9.2 | 0.61 |
| Ischaemia present (%) | 10/49 (20.4) | 5/23 (21.7) | 0.90 |
| Ischaemic burden (%) in patients with ischaemia | 13.1±10.5 | 16.9±13.5 | 0.56 |
| Ischemic burden > 20% | 3/49 (6.1%) | 2/23 (8.7%) | 0.69 |

Data presented as n/N (%), mean ±SD or median (IQR). CR= complete revascularization; IRA= Infarct related artery; LVMI= left ventricular mass index; LVEDVI= left ventricular end-diastolic volume index; LVEF= left ventricular ejection fraction; LGE= late gadolinium enhancement; IS=infarct size; MVO= microvascular obstruction; MSI= myocardial salvage index

^§^ Analyzable oedema imaging available in 76% of patients in both groups.

* Adjusted for known predictors of IS (anterior MI, time to revascularization, diabetes, TIMI flow pre-PPCI) and important baseline variables significantly varying between the two groups (TIMI flow post-PPCI, SYNTAX score, dual antiplatelet therapy choice, glycoprotein inhibitor/bivalirudin use for N-IRA PCI)

Supplemental Table 3: Discharge medication

| **Variable** | **One-time CR (n=63)** | **Staged CR (n=30)** | **p** |
| --- | --- | --- | --- |
| Beta-blocker (n, %) | 61 (96.8) | 27 (90.0) | 0.17 |
| ACEI or ARB(n, %) | 58(92.1) | 29 (96.7) | 0.40 |
| Statin (n,%) | 60 (100) | 30 (100) | 1.00 |
| Loop diuretic (n, %) | 5 (7.9) | 3 (10) | 0.74 |
| Aldosterone antagonist (n,%) | 3 (4.8) | 2 (6.7) | 0.75 |

ACEI= angiotensin converting enzyme inhibitor; ARB= angiotensin receptor blocker

**Supplemental Table 4: Clinical outcomes**

| **Variable** | **Immediate CR**  **(n=63)** | **Staged CR**  **(n=30)** | **HR (95% CI)** | **p** |
| --- | --- | --- | --- | --- |
| **12 month follow-up** |  |  |  |  |
| MACE (n, %) | 4/63 (6.3) | 2/30 (6.7) | 1.05 (0.18, 6.1) | **0.97** |
| Death (n, %) | 0/63 (0.0) | 1/30 (3.3) | ** | 0.15 |
| Recurrent MI (n, %) | 0/63 (0.0) | 0/30 (0.0) | ** | 1.00 |
| Heart failure (n, %) | 2/63 (3.2) | 1/30 (3.3) | 0.97 (0.09, 12.1) | 0.97 |
| Revascularisation (n, %) | 2/63 (3.2) | 0/30 (0.0) | ** | 0.32 |
| **Inpatient clinical events** |  |  |  |  |
| Death (n, %) | 1/63 (1.6) | 0/30 (0.0) | ** | 0.49 |
| Recurrent MI (n, %) | 0/63 (0.0) | 0/30 (0.0) | ** | 1.00 |
| Heart failure (n, %) | 2/63 (3.2) | 0/30 (0.0) | ** | 0.32 |
| Repeat revascularisation (n, %) | 1/63 (1.6) | 0/30 (0.0) | ** | 0.49 |
| **Safety Endpoints** |  |  |  |  |
| Contrast nephropathy (n, %) | 1/63 (1.6) | 0/30 (0.0) | ** | 0.49 |
| Vascular access injury (n, %) | 0/63 (0.0) | 0/30 (0.0) | ** | 1.00 |
| CVA/TIA (n, %) | 0/63 (0.0) | 0/30 (0.0) | ** | 1.00 |
| Major bleed (n, %) | 0/63 (0.0) | 3/30 (10.0) | ** | 0.011 |

CR= complete revascularization; IRA= Infarct related artery; MACE= major adverse cardiovascular events, HF= heart failure. ** As there 0 events in at least one of the groups HR calculation is not possible.
